# Supplementary figures and images for: Transcriptomic analysis reveals pronounced changes in gene expression due to sub-lethal pyrethroid exposure and ageing in insecticide resistance Anopheles coluzzii
Source: BMC Genomics. 2021 May 10;22:337. doi: 10.1186/s12864-021-07646-7 (PMC8111724; doi:10.1186/s12864-021-07646-7)

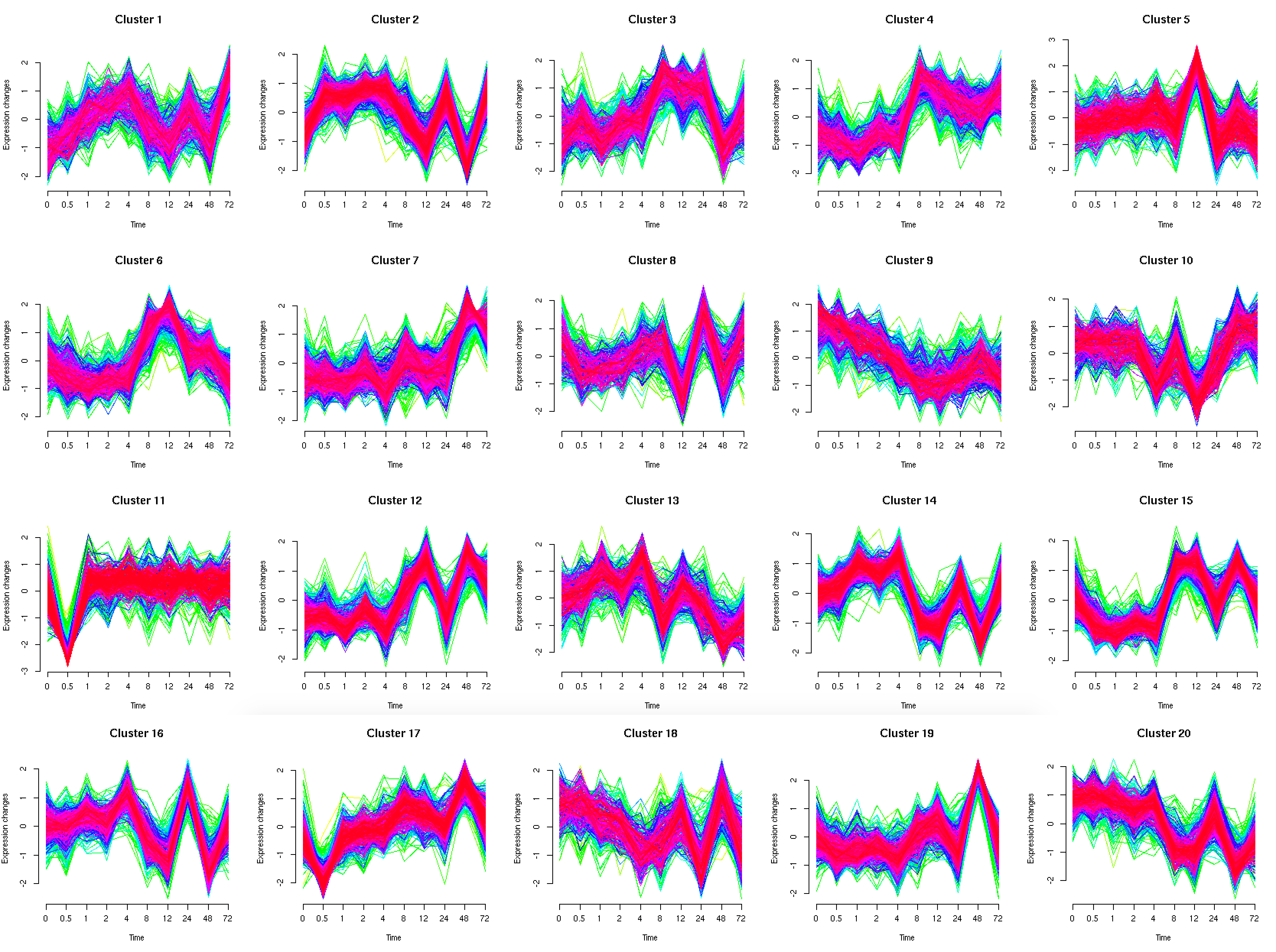

Supplement: Supplementary file 1 — Additional file 1. Mfuzz clusters for all transcripts. Expression patterns for all transcripts across the 20 soft Mfuzz clusters. Red indicated high cluster membership, blue intermediate and green low. Y-axis indicated normalised expression change and the x-axis represents each time point. [file 12864_2021_7646_MOESM1_ESM.png]

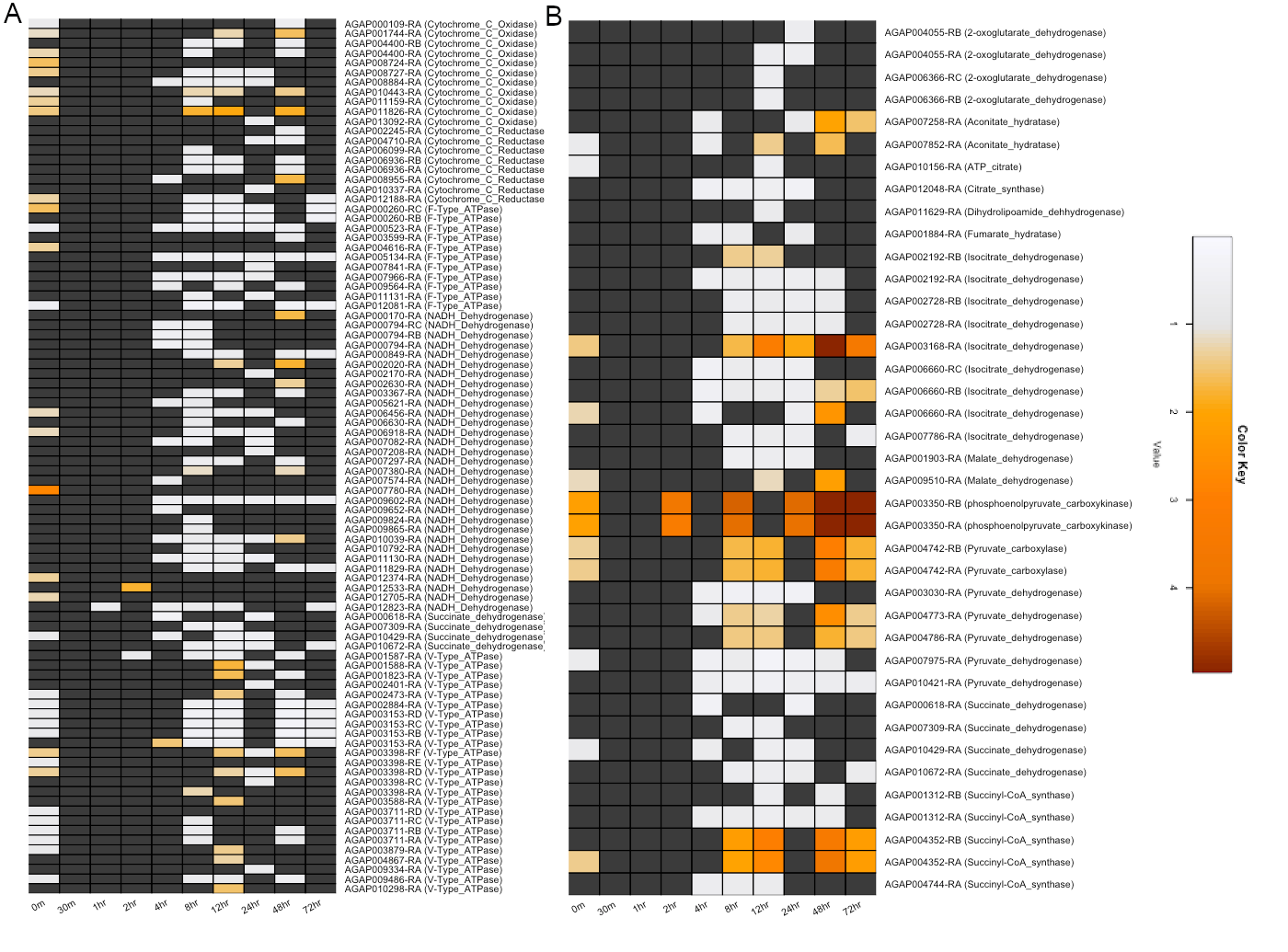

Supplement: Supplementary file 8 — Additional file 8. Significant changes in respiratory-related transcripts. Heatmaps showing transcripts involved in (A) Oxidative phosphorylation and (B) TCA cycle that are differential in at least one time point. Pathway membership as defined by KEGG. Transcript ID followed by generic name is shown in row labelling, columns represent different timepoints. Dark grey indicated non-significant. [file 12864_2021_7646_MOESM8_ESM.png]

A

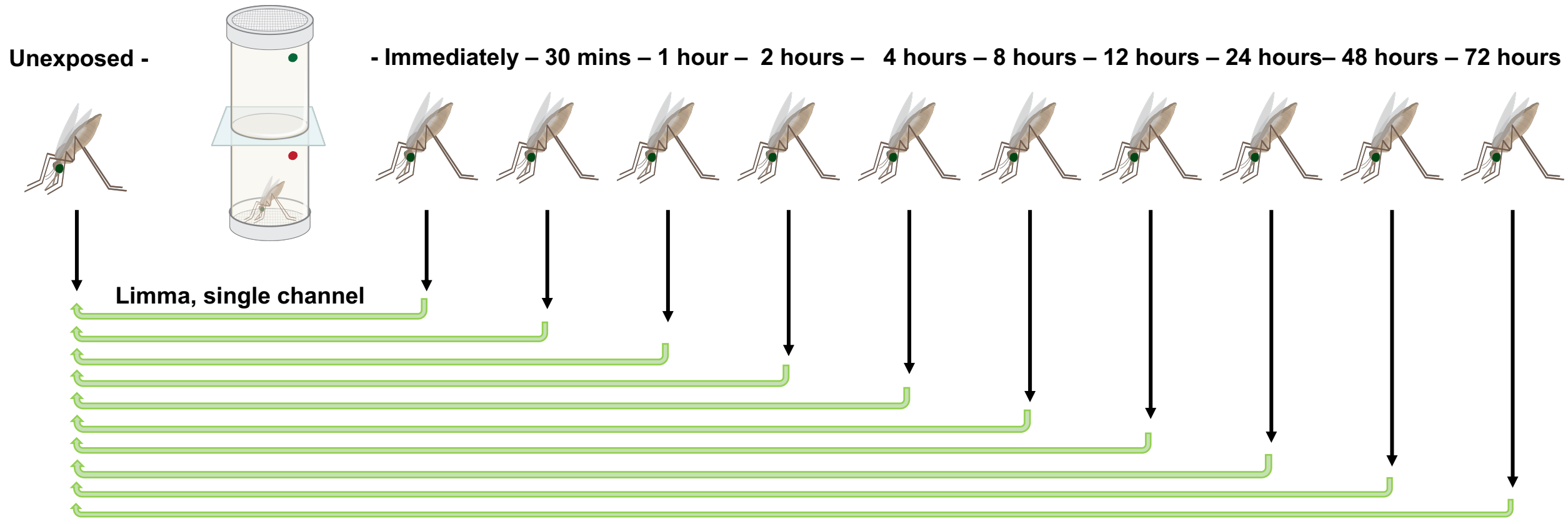

B

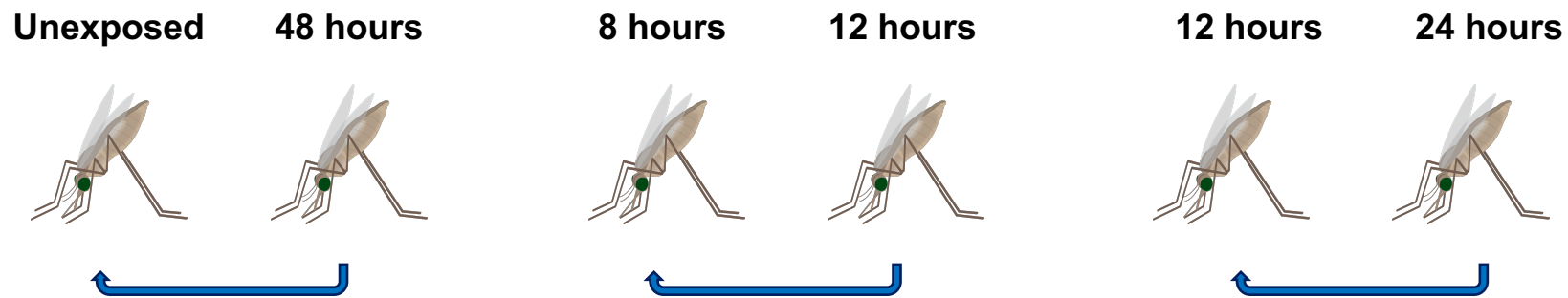

Supplement: Supplementary file 11 — Additional file 11. Experimental Design. A. Exposure time course, black arrows represent the time at which mosquitoes were harvested for RNA extraction. In the original experimental design, each array was hybridised to the time point before (hyphens). For this paper, limma single channel analysis was used to compare each time point with an unexposed time point (green arrows). WHO tube shows point of exposure. B. Unexposed dataset, dark blue arrows represent direct array hybridisation. [file 12864_2021_7646_MOESM11_ESM.pdf]
